# Middle school cycling program is associated with improved mental health and wellbeing in adolescents during COVID-19

**DOI:** 10.3389/fspor.2023.1255514

**Published:** 2023-10-12

**Authors:** Fletcher Dementyev, Brian Fish, Nana Yaa Sakyi Opoku, Lydia Tesfaye, Jason Chan, Larry Ortiz, Susanne B. Montgomery, Esther J. Walker, Sean M. Wilson

**Affiliations:** ^1^ Lawrence D Longo Center for Perinatal Biology, Loma Linda University School of Medicine, Loma Linda, CA, United States; ^2^ Department of Social Work and Social Ecology, Loma Linda University School of Behavioral Health, Loma Linda, CA, United States; ^3^ Behavioral Health Institute, Loma Linda University School of Behavioral Health, Loma Linda, CA, United States; ^4^ Outride, Morgan Hill, CA, United States

**Keywords:** COVID-19, cycling, depression, WHO-5, PSC-17-Y

## Abstract

**Introduction:**

The COVID-19 pandemic has exacerbated mental health issues among adolescents. Exercise is well-regarded for boosting mental health. Riding for Focus (R4F) is a 6–8 week cycling education program designed to equip middle school adolescents with basic cycling skills and introduce students to lifetime physical activity. A secondary goal of the R4F program is to improve adolescent mental health and psychosocial well-being. This study aimed to quantify the change in adolescent psychosocial well-being associated with the R4F program during the COVID-19 pandemic. Program evaluation also examined associations between participating in the R4F and mental health outcomes in the context of established risk factors, including gender, race, socioeconomic status, involvement in IEP programs, participation in after-school clubs, screen time, hours of sleep, and physical activity levels.

**Methods:**

Anonymous surveys were collected before and after the program in 20 schools in North America, with psychosocial well-being quantified using WHO-5 and PSC-17-Y. 1,148 middle school students, aged 11–14, completed pre intervention surveys. 815 students also completed post intervention surveys.

**Results:**

There was a general increase in psychosocial well-being after the R4F program and positive psychosocial well-being changes in students that identified as female, non-white, physically active, part of an IEP program, meeting screentime recommendations, and engaged in school programs, though effect sizes were small. Despite mental health improvements among underrepresented groups, relative risk assessments still indicated that males, white students, those from high socioeconomic status families still had reduced relative risk of developing psychosocial disorders post intervention.

**Discussion:**

These analyses illustrate the feasibility of cycling as a viable PE elective and the need for further, more robust studies to better assess the positive impacts of the R4F scholastic cycling program on the psychological health and well-being of middle school age children.

## 1. Introduction

The mental well-being of school-aged children continues to deteriorate in the United States. One in six school-aged children are diagnosed with mental health disorders (1); 13% of whom experience major depressive episodes (2). The COVID-19 pandemic further contributed to a deterioration in the mental health of school aged children, which has led to the 2021 surgeon general advisory on a mental health crisis in youth (3). Rates of depression, anxiety, and post-traumatic stress disorder symptoms increased in high school students due to COVID-19 lockdowns (4). The positive impact of physical activity on mental, physical, and cognitive health is well documented (5–7). Unfortunately, physical activity rates among youth continue to decrease, particularly among those from low socioeconomic status (SES) backgrounds. COVID-19 lockdowns further decreased physical activity among all age groups and this decrease in exercise was associated with an increase in levels of depression and anxiety (8).

Data from the National Center for Education Statistics shows that adolescents in the United States spend an average of 6.64 h in class, and that they are in school for roughly 180 days each year (9). Because they are in school for an appreciable amount of time, introducing students to the many benefits of exercise through physical education courses is important. School physical education programs help youth work towards their daily recommended movement goals and more broadly, to make positive lifestyle choices. Physical education classes provide an environment where kids can participate in physical activity with their friends. Youth that participate in physical activity with their friends are much more likely to be motivated to be physically active (10), thus exerting a positive peer influence. Unlike intramural or extramural sports opportunities, where significant socioeconomic disparities affect participation, in-school physical education provides every student opportunities to learn about and engage in physical activity (11). Unfortunately, less than 4% of K-12 schools require daily physical education or its equivalent (12) and not all physical education initiatives result in positive outcomes. Students may actively avoid physical education, resulting in negative health and educational consequences (13). These findings illustrate that not all physical education initiatives are created equal, which makes it important to understand what types of physical education initiatives excite and motivate students to participate. Recent evidence suggests that the most successful physical education initiatives motivate and engage students with fun and social activities (11). Thus, if offered in an engaging fashion, Physical Education programs have the potential to help schools harness the enthusiasm most students have about returning to school after being out on holiday breaks or learning remotely due to the COVID-19 pandemic.

One fun and engaging physical activity that gained popularity during the COVID-19 pandemic is cycling. Introducing cycling is a promising approach to engage students in physical activity during the school day (14). Cycling is a unique physical activity as bicycles can be used for transportation, exploration, recreation, health, wellness, as well as competition and can be performed alone or with friends. The bicycle therefore provides many advantages for building and enjoying a physically active lifestyle as compared to other endurance activities (e.g., swimming) and sports (e.g., soccer) that are performed in facilities or highly structured team atmospheres with performance expectations. Associated with this, people who ride bikes are physically healthier (15). Riding bikes also engenders feelings of joy (16, 17), decreases incidents of mental distress (18), and increases a sense of community attachment and social inclusion (15). Cycling also provides occasions to spend much needed time in greenspaces, which increases mental well-being (19).

Programs centered around the bicycle therefore afford opportunities and benefits that many other physical activity programs cannot. One such cycling program is the Outride Riding for Focus (R4F) program. The R4F program is a 6–8 week middle school physical education program with a curriculum designed to equip students with basic cycling handling skills and introduce them to the lifetime physical activity of cycling. Current literature indicates that physical activity interventions have the ability to positively increase adolescent mental health and psychosocial well-being (20, 21). Therefore, the expectation is that introducing a school-based cycling intervention will result in improved behavioral health and well-being of adolescents in middle schools, primarily aged 10–14. We hypothesize that students participating in the R4F program will have improvements in student psychosocial well-being. This hypothesis was explored by having student participants take electronically administered surveys with validated measures of psychosocial well-being before and after participation in the R4F program.

## 2. Methods

### 2.1. Participants and methods

Data for this program evaluation study were collected via a voluntary anonymous survey from participants in the Outride R4F scholastic middle school cycling physical education program. Student participants filled out online surveys via the Qualtrics platform on classroom or at-home electronic devices before and after the R4F program. Institutional review board approval was obtained by IntegReview (now Advarra) and for secondary data analysis by the institutional review board of Loma Linda University. The data were collected from 1,268 pre-program respondents and 910 post-program respondents from 20 middle schools in North America participating in the R4F program from January–May 2021. Students were between the ages of 11 and 14, as commensurate with their grade level (6–8). Schools included in this study were from a mix of urban, suburban, and rural regions throughout the United States. There was roughly an equal proportion of 57% male and 43% female respondents before and after the program. 64% of respondents self-identified as white, while 36% of respondents self-identified with other racial or ethnic groups (American Indian and Alaska Native, Asian, Black or African American, Hispanic or Latina/Latino, Native Hawaiian and Other Pacific Islander, Two or More Races), consistent with the demographics of the participating schools. There was not enough statistical power to measure differences between all racial groups and as a result, for analyses reported here, we report differences between participants who self-identified as white and those who self-identified as a different racial or ethnic background (non-white). Despite COVID-19, 45 schools implemented the R4F program in the 2020–2021 school year; thus, our sample represents 44% of the total number of schools who were able to implement the program during that school year.

Students were asked to take a survey before the R4F program through a Qualtrics online portal, which included questions about student experiences and perceptions of physical activity, cycling, school, and overall well-being. Students also answered questions about hours of sleep, hours of screen time, level of physical activity, and involvement in clubs and sports teams. Students self-reported their gender, race and ethnicity, whether they previously participated in a R4F program, and whether they received free or reduced-cost school lunches, which was used as a proxy for socioeconomic status (SES). They were also asked whether they were enrolled in an Individualized Education Plan (IEP), which is a legal plan or program developed to ensure that a child with an identified disability receives educational accommodations that ensure educational performance in elementary and secondary school (22). Students also completed two assessments to measure psychosocial wellbeing. This included the WHO-5 Well-Being Index, which is a short, 5 item, self-report questionnaire that assesses positive well-being and primarily assesses for depression. WHO-5 has been validated with adolescent samples across the world, where higher scores indicate greater well-being (23, 24). Participants also completed the youth self-report version of the Pediatric Symptom Checklist (PSC-17-Y) (25), which is a widely used measure of psychosocial functioning, with lower scores representing greater well-being (26). The PSC-17-Y helps identify and assess changes in emotional and behavioral problems and covers a range of emotional and behavioral problems and assesses psychosocial functioning. The PSC-17-Y also has sub-scores reflecting externalization (incudes conduct, oppositional defiant disorder, adjustment disorder with disturbed conduct or mixed disturbed mood and conduct), internalization (any anxiety and mood disorder), and attention (ADHD and ADD), which were also examined individually in the current study. Students then took an identical survey after the R4F program with the addition of open and closed questions about the R4F program. The survey was designed to be completed in 10–15 min.

Due to COVID-19 school closures, not every student was able to complete both a pre-and post-program survey. The number of respondents for the pre-survey was 1,268 and 910 for the post-survey. The mean time to completion of the survey was 11.6 min, with a median of 9.3 min. Surveys were removed if they were completed in fewer than 3 min or were not completed within 2 standard deviations longer than the mean completion time (2.77 h). A secondary filter was performed to ensure the quality of the PSC-Y-17 and the WHO-5 responses. Only those responses that included a response to each sub-item were included to ensure scores were not skewed by non-responses to individual items. This final filter resulted in the removal of 8.1% of PSC/WHO responses, with 1,148 pre-program and 815 post-program surveys being analyzed.

### 2.2. Cycling program curriculum

The Riding for Focus (R4F) program is a middle school cycling education program designed by the organization Outride. The program is designed to teach cycling safety skills so that participants can cycle competently, efficiently, and effectively Students learn how to form an exercise habit, enjoy exercising, and how exercise can be used to improve other aspects of their lifestyle. The program was developed based on principles of effective instruction, program design, and evaluation and was motivated by Social Cognitive and Self-Determination theories. The curriculum was designed to be taught in chronological, developmentally progressive order, ultimately leading teachers and students through a structured, repetitive, and iterative 12 lesson process. The key lesson topics covered are listed in Table 1. These lessons are taught over 18–24 class sessions and 13–18 contact hours, providing students with regular cycling experience, allowing students to develop their cycling skills and more broadly to experience self-determination and autonomy with cycling. The program follows the PHS guidelines for physical activity (27), and those of SHAPE America (28).

**Table 1:** Riding for focus lesson topics.

Lesson number	Lesson topic
Lesson 0	Program Introduction
Lesson 1	Bike, Helmet, and Routines
Lesson 2	Pre-Ride Check and Start
Lesson 3	Stop and Balance
Lessons 4a and 4b	Maintaining Control; Cadence and Shifting
Lesson 5	Heart Rate and Training Zones
Lesson 6	Weaving through Obstacles
Lesson 7	Turning Skills and Scanning
Lesson 8	Hand Signals, Advanced Scanning, and Anticipation
Lesson 9	Managing Hazards
Lesson 10	Traffic Safety and Cycling Etiquette
Lesson 11	Cycling Safely
Lesson 12	Road Readiness

The R4F program was designed to be implemented during the school day, often during a Physical Education class, for a minimum of three days a week for at least six weeks where possible, with a focus on achieving at least 20 min of moderate-to-vigorous physical activity during each class session. The lessons can be taught on a wide range of terrains and settings including basketball or tennis courts, track fields, empty parking lots, or a grass field.

### 2.3. Statistical methods

Data analysis and the production of graphs was performed using GraphPad Prism 9.1.2 (La Jolla, CA). Data was tested for normality prior to analysis. None of the data was normally distributed. For analyses between two groups, a Mann–Whitney *U* test was used to identify potential differences in the well-being metrics on non-parametric datasets. For analysis of more than two groups, a Kruskal-Wallis ANOVA with a Dunn's multiple comparisons test was performed on non-parametric datasets. Graphs present violin plots with median, 25% and 75% quartiles, along with minimum and maximum data ranges. A Chi square analysis was performed to examine the proportion of students in each group before and after the program. A *P*-value of *P* < 0.05 was considered significant and was further broken down to *P* < 0.01, *P* < 0.001, and *P* < 0.0001 where appropriate. Additionally, Cohen's *d* test statistic was calculated to approximate effect size in pre and post intervention comparisons. Based on benchmarks recommended by Cohen, interpretations of effect size included small (*d *= .2), medium (*d *= .5) and large (*d* = .8) (29). Because the PSC-17-Y and WHO-5 are used for clinical diagnosis we examined the relative clinical risk of developing disorders using a Fisher Exact Test in combination with Koopman asymptotic scores (30) for the primary effect and sub-groups of students who met or did not meet critical cutoff criteria for WHO-5 and PSC-17-Y, including gender, SES, race, IEP program, physical activity, sleep, screen time per day, and involvement in school programs. The cutoff for physical activity was 4 days a week of 60 min of exercise, as adolescents who do not exercise at least 4 days a week for 60 min have an increased risk for the development of psychosocial disorders compared to those who do exercise 4 days or more (31). The cutoff for sleep was 8.5 h of sleep each weeknight as the available evidence suggests that adolescents who sleep less than 8.5 h a night on school nights are at greater risk of psychosocial disorders than those who do meet the 8.5-hour threshold (32). The critical cutoff for screen time was determined to be 2 h a day, as recommended by the American Academy of Pediatrics' 2016 policy statement on adolescent media use (5). The WHO-5 and PSC-17-Y are both validated metrics of psychosocial well-being with established cutoff scores of ≤50 and ≥15, respectively, that indicate high risk for psychological impairment (24, 26).

## 3. Results

**Figure 1:**
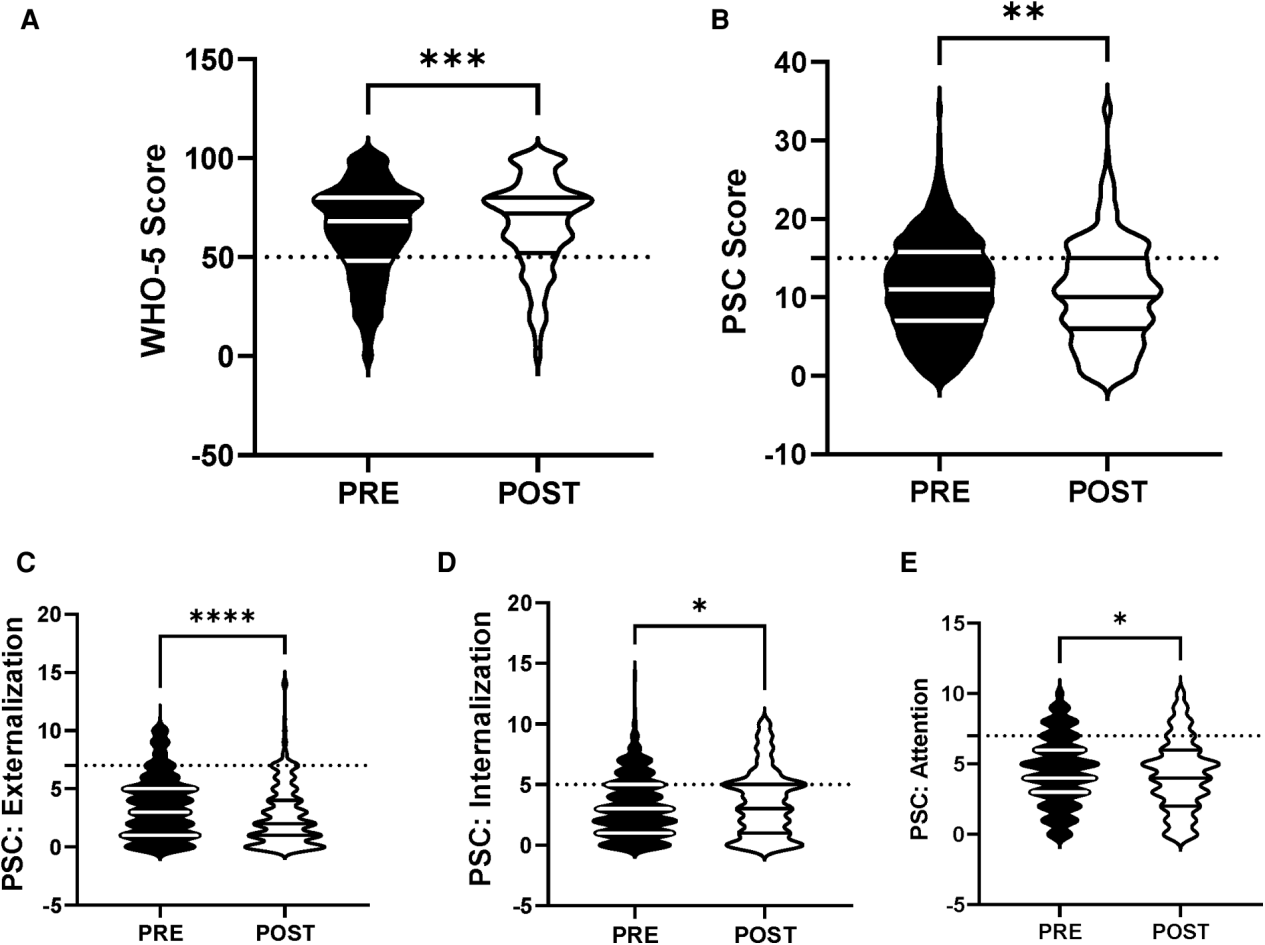
General effectiveness of the R4F program on psychosocial wellness. (**A**) Distribution of WHO-5 well-being scores in a violin plot. Dashed horizontal line shows the critical risk cutoff of 50 points with lower scores indicating lower well-being. (**B**) Distribution of PSC-17-Y composite well-being scores with a critical risk cutoff of 15 points. (**C–E**) PSC-17-Y subscores of attention, internalization, or externalization on violin plots with critical risk cutoffs of 7 points, 5 points, and 7 points, respectively. Higher scores indicate lower well-being. Within each violin plot the median and 25% and 75% quartiles are shown. Asterisks denote significance difference between groups based on a Mann—Whitney *U* test (*<0.05, **<0.01, ***<0.001, ****<0.0001). *N* = 1,148 for pre-program, *N* = 815 for post-program.

The efficacy of the R4F program in improving mental health and well-being was evaluated for a primary effect of the program in all students. Following the R4F program there was a significant increase in the WHO-5 composite score and a significant decrease in PSC-17-Y composite score post-program (Figures 1A,B). There was also a significant decrease in PSC-17-Y sub-scores for externalization, internalization, and attention following the program (Figures 1C,D,E). Before the R4F program 30% (347) of the 1,148 adolescent subjects were found to have PSC-17-Y scores ≥15, while after the program this was not significantly different with 27% (221) students having scores ≥15. With regards to the WHO-5 metric, 26% (300) students had scores ≤50 before the R4F program and significantly fewer 21% (173) had scores ≤50 after the program.

### 3.1. What factors influence well-being, and do they interact with participation in R4f?

#### 3.1.1. Gender

**Figure 2:**
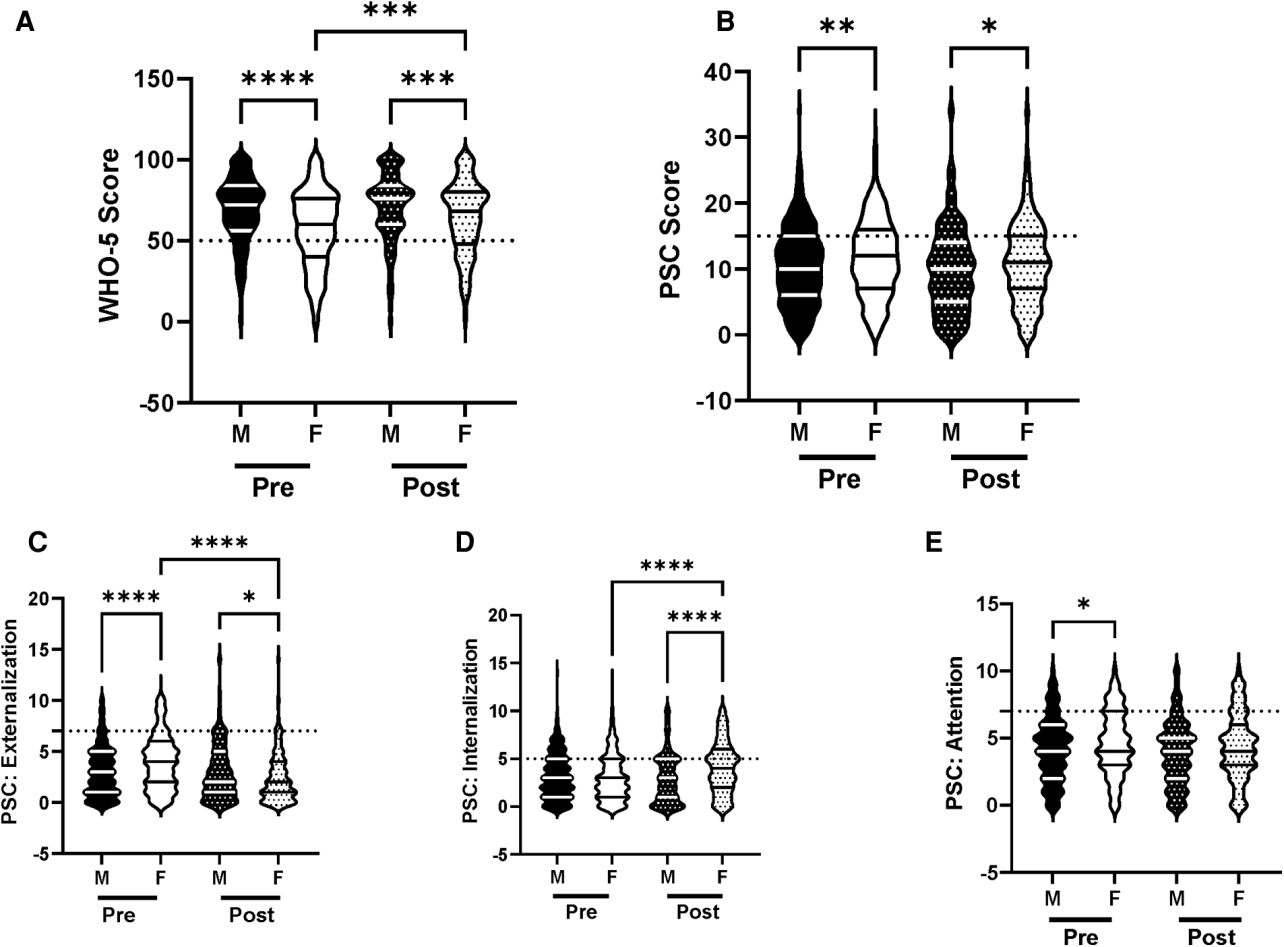
Impacts of gender on psychosocial wellness and effectiveness of the R4F program. (**A**) Distribution of WHO-5 well-being scores in a violin plot. Dashed horizontal line shows the critical risk cutoff of 50 points with lower scores indicating lower well-being. (**B**) Distribution of PSC-17-Y composite well-being scores with a critical risk cutoff of 15 points. (**C–E**) PSC-17-Y subscores of attention, internalization, or externalization on violin plots with critical risk cutoffs of 7 points, 5 points, and 7 points, respectively. Higher scores indicate lower well-being. Within each violin plot the median and 25% and 75% quartiles are shown. Asterisks denote significance difference between groups based on a Kruskal-Wallis One-way ANOVA with a Dunn’s multiple comparison test based on ranks (*<0.05, **<0.01, ***<0.001, ****<0.0001). Pre-program, *N* = 611 for male, *N* = 468 for female. Post-program, *N* = 424 for male, *N* = 335 for female.

We analyzed the efficacy of the R4F program in male and female adolescent students to examine the influence of gender, as gender related differences in psychosocial well-being and responses to exercise programs are well documented (18, 33–35). WHO-5 composite scores were significantly higher (Figure 2A) while PSC-17-Y composite scores were significantly lower (Figure 2B) in males compared to females before students participated in the R4F program. Before participating in the R4F program externalization and attention scores were higher in females (Figures 2C,E) while internalization was equivalent between male and female students (Figure 2D). Before participating in the R4F program female students had an increased risk of developing psychosocial disorders as measured by either WHO-5 or PSC-17-Y (Figures 10A,B).

There were differences in how each gender responded to the R4F cycling program. Participation in the R4F program was associated with a significant improvement in WHO-5 psychosocial well-being scores in female students but not their male counterparts (Figure 2A). However, males still had higher WHO-5 scores as compared to females after the R4F program (Figure 2A). Participation in the R4F program was not associated with an improved PSC-17-Y score in either male or female students (Figure 2B), with males continuing to have significantly lower PSC-17-Y scores as compared to females. Program participation was associated with lower externalization sub-scores for females (Figure 2C) and elevation in internalization (Figure 2D) without any changes in the sub-scores for males (Figures 2C,D,E). Following the R4F program the mean relative risk for developing a psychological disorder improved for females from 28% to 14% as measured with WHO-5 while the PSC-17-Y was unchanged, although the relative risk for females remained higher than males (Figures 10A,B). The proportion of males who took the survey was unchanged before (57%) and after (56%) the R4F program.

#### 3.1.2. Race and ethnicity

**Figure 3:**
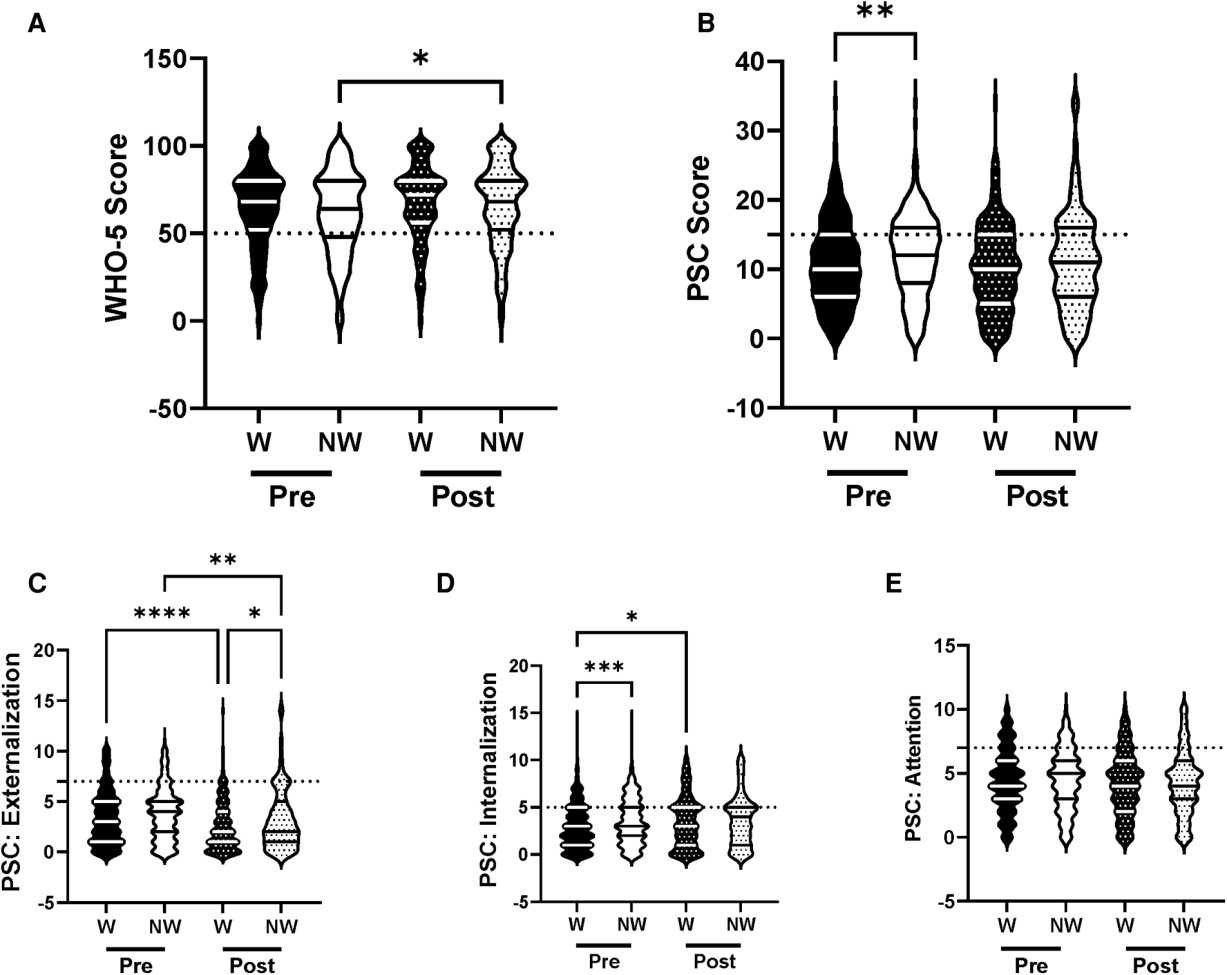
Impacts of racial identity on psychosocial wellness and effectiveness of the R4F program. (**A**) Distribution of WHO-5 well-being scores in a violin plot. Dashed horizontal line shows the critical risk cutoff of 50 points with lower scores indicating lower well-being. (**B**) Distribution of PSC-17-Y composite well-being scores with a critical risk cutoff of 15 points. (**C–E**) PSC-17-Y subscores of attention, internalization, or externalization on violin plots with critical risk cutoffs of 7 points, 5 points, and 7 points, respectively. Higher scores indicate lower well-being. Within each violin plot the median and 25% and 75% quartiles are shown. Asterisks denote significance difference between groups based on a Kruskal-Wallis One-way ANOVA with a Dunn's multiple comparison test based on ranks (*<0.05, **<0.01, ***<0.001, ****<0.0001). W, White students; NW, non-white students. Pre-program, *N* = 733 for white students, *N* = 415 for non-white students. Post-program, *N* = 500 for white students, *N* = 315 for non-white students.

To examine the influence of race and ethnicity we analyzed the efficacy of the R4F program for both white and non-white students, because previous evidence indicates that minorities report lower levels of psychosocial well-being (36). There were no baseline differences in WHO-5 composite well-being scores although PSC-17-Y composite scores were increased in non-white students (Figures 3A,B). Internalization was also increased in non-white relative to white students (Figure 3D), while externalization and attention were equivalent. Secondarily, there was an increased risk of developing psychosocial disorders in non-white students at baseline as measured with WHO-5 and PSC-17-Y (Figures 10A,B).

Following the R4F program there was a significant increase in the WHO-5 well-being composite scores of non-white students but no differences in white students. No significant differences were observed in white and non-white students' well-being scores in the PSC-17-Y metrics following program participation (Figure 3B). However, the PSC-17-Y sub-scores revealed that the R4F program was associated with decreased externalization scores of white and non-white students, and internalization scores of white students (Figures 3C,D). Following the R4F program externalization scores were significantly higher in non-white students than their white counterparts (Figure 3C). The R4F program was not associated with any change in attention (Figure 3E). The relative risk for the development of psychosocial disorders of non-white students were improved and equivalent to white students following the R4F program based on both WHO-5 and PSC-17-Y (Figures 10A,B). The proportion of adolescents who were white was equivalent before (64%) and after (61%) the R4F program.

#### 3.1.3. Socioeconomic Status

**Figure 4:**
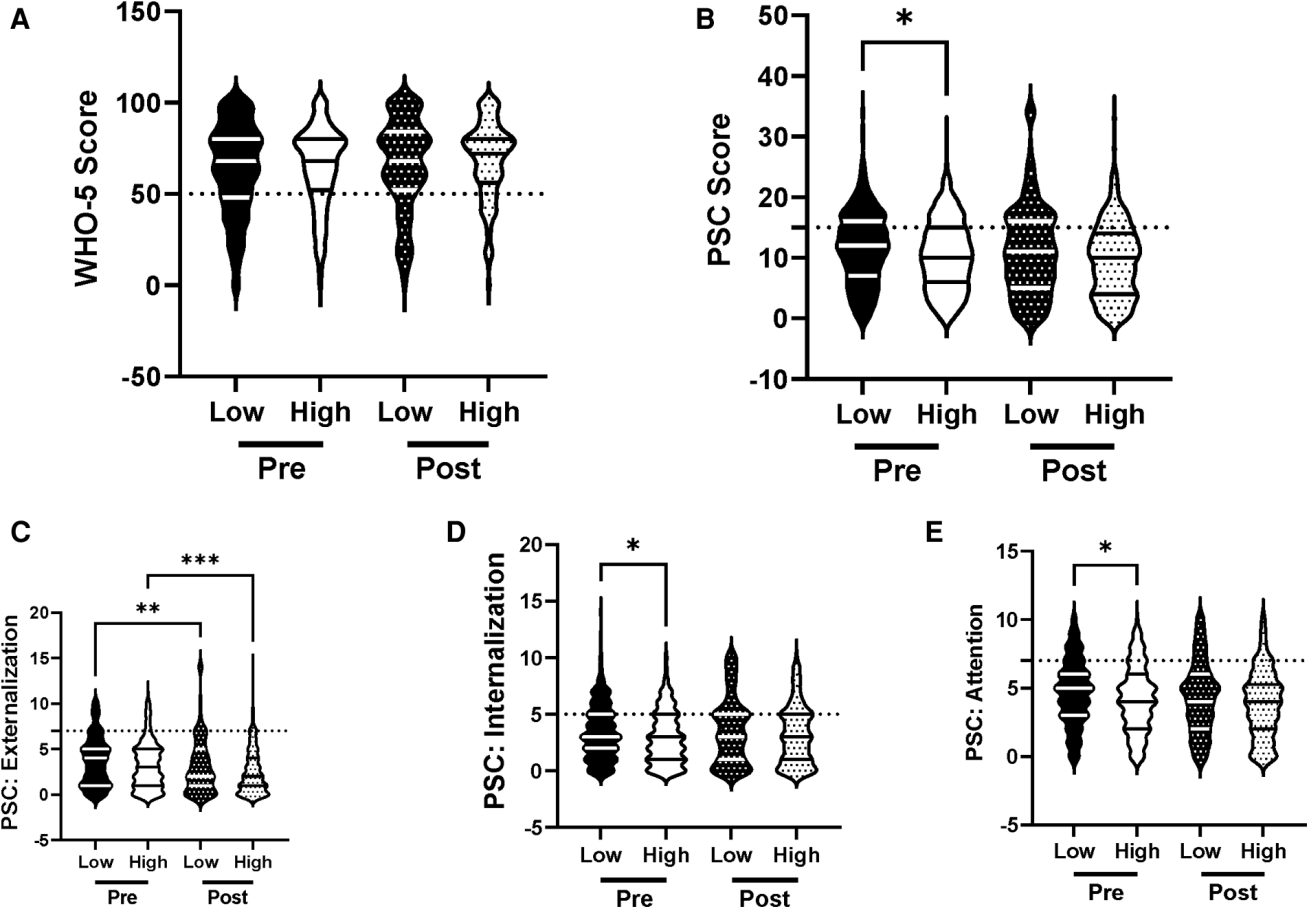
Impacts of socioeconomic status on psychosocial wellness and effectiveness of the R4F program. (**A**) Distribution of WHO-5 well-being scores in a violin plot. Dashed horizontal line shows the critical risk cutoff of 50 points with lower scores indicating lower well-being. (**B**) Distribution of PSC-17-Y composite well-being scores with a critical risk cutoff of 15 points. (**C–E**) PSC-17-Y subscores of attention, internalization, or externalization on violin plots with critical risk cutoffs of 7 points, 5 points, and 7 points, respectively. Higher scores indicate lower well-being. Within each violin plot the median and 25% and 75% quartiles are shown. Asterisks denote significance difference between groups based on a Kruskal-Wallis One-way ANOVA with a Dunn's multiple comparison test based on ranks (*<0.05). H, intermediate/high SES; L, low SES. Pre-program *N* = 336 for low SES, *N* = 391 for intermediate/high SES. Post-program, *N* = 251 for low SES, *N* = 270 for intermediate/high SES.

We analyzed the efficacy of the R4F program within the context of socioeconomic status (SES), a demographic factor that is known to increase risk for adolescent psychosocial well-being (37). Baseline data revealed that students who qualified for free or reduced fee lunches had equivalent WHO-5 scores (Figure 4A) but higher PSC-17-Y composite scores (Figure 4B). These students also had equivalent sub-scores for externalization (Figure 4C) but increased sub-scores for internalization, and attention (Figures 4D,E). Before the R4F program students who qualified for free or reduced fee lunches also had increased risk of the development of psychosocial disorders as measured by PSC-17-Y but not WHO-5 (Figures 10A,B).

Participation in the R4F program had no effect on WHO-5 (Figure 4A) but was associated with an equalization in PSC-17-Y between students who qualified or did not qualify for free or reduced fee lunches (Figure 4B). Externalization was improved in students who qualified or did not qualify for free or reduced fee lunches (Figure 4C) while internalization and attention were equivalent in the two groups of students (Figures 4D,E). The risk of developing psychosocial disorders between the two SES groups as determined with WHO-5 was unaffected by the R4F program (Figure 10A) while the mean relative risk as determined by PSC-17-Y decreased from 22% to 13% but was not completely normalized (Figures 10A,B). The percentage of adolescents who were not eligible for school lunch programs was equivalent before (54%) and after (52%) the R4F program.

#### 3.1.4. Individualized education plan

**Figure 5:**
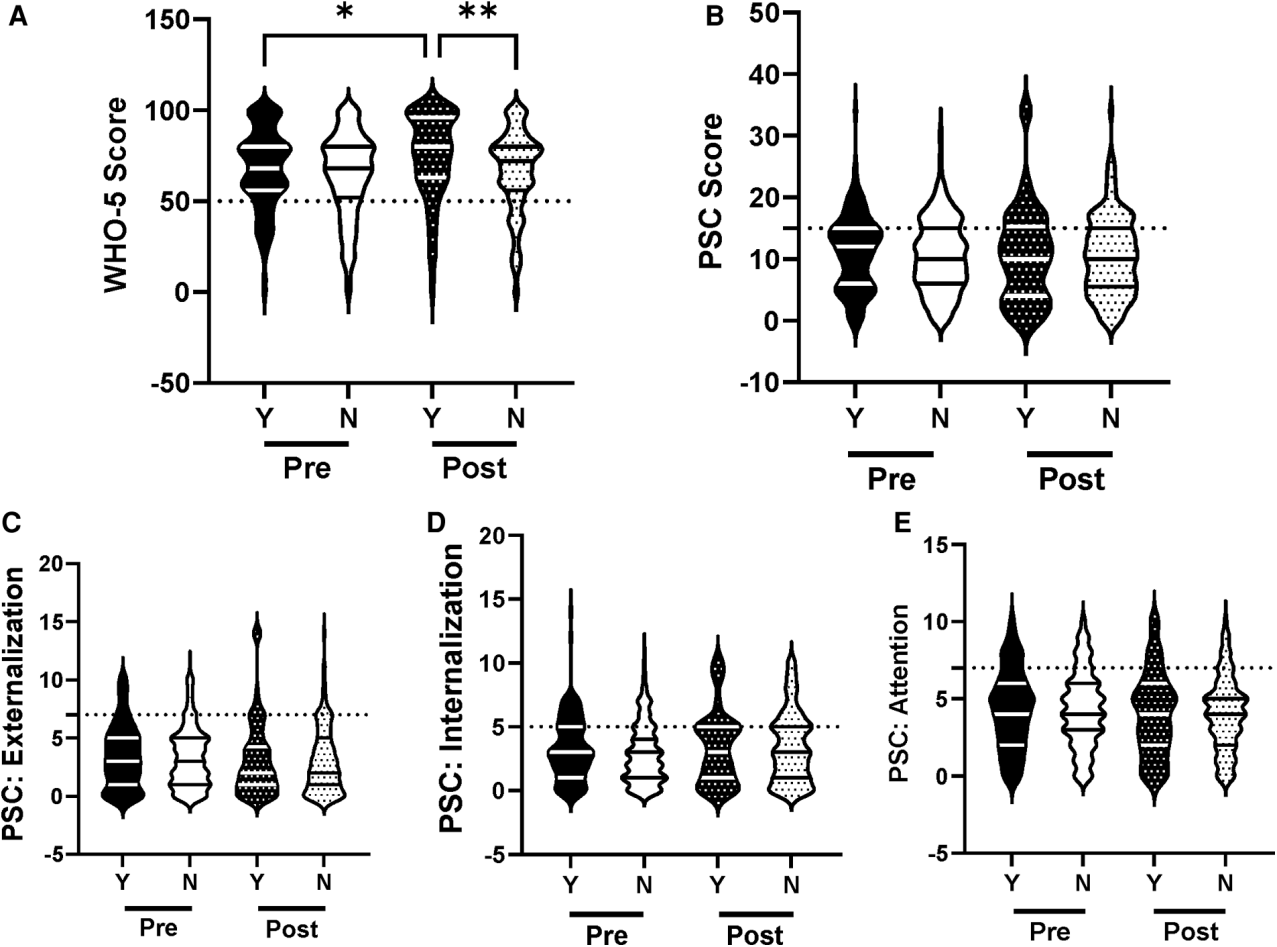
Impacts of having an IEP on psychosocial wellness and effectiveness of the R4F program. (**A**) Distribution of WHO-5 well-being scores in a violin plot. Dashed horizontal line shows the critical risk cutoff of 50 points with lower scores indicating lower well-being. (**B**) Distribution of PSC-17-Y composite well-being scores with a critical risk cutoff of 15 points. (**C–E**) PSC-17-Y subscores of attention, internalization, or externalization on violin plots with critical risk cutoffs of 7 points, 5 points, and 7 points, respectively. Higher scores indicate lower well-being. Within each violin plot the median and 25% and 75% quartiles are shown. Asterisks denote significance difference between groups based on a Kruskal-Wallis One-way ANOVA with a Dunn's multiple comparison test based on ranks (*<0.05, **<0.01). Y, students with an IEP program; N, students who do not have an IEP program. Pre-program, *N* = 126 for IEP, *N* = 359 for no IEP. Post-program, *N* = 102 for IEP, *N* = 281 for no IEP.

We analyzed the influence of having an Individualized Education Plan (IEP) on the efficacy of the R4F program to increase the psychosocial well-being and mental health of adolescents as the available evidence demonstrates that adolescents with IEPs are at increased risk for an array of mental health challenges (38, 39). Analysis of pre-program surveys revealed no baseline differences in WHO-5 and PSC-17-Y well-being scores between students with IEPs and those without (Figures 5A,B). However, participation in the R4F program was associated with a significant increase in the WHO-5 scores of students with IEPs, resulting in students with IEPs having significantly greater WHO-5 scores than those without IEPs. This improvement in WHO-5 scores was not replicated by the PSC-17-Y composite well-being scores. The PSC-17-Y sub-scores of attention, internalization, or externalization were equivalent between students with or without IEP plans and there were not influenced by the R4F program (Figures 5C,D,E). Students with or without IEP plans had similar relative risk for the development of psychosocial disorders before as well as after the R4F program (Figures 10A,B). The percentage of adolescents with IEP programs was unchanged pre and post survey, being 26% before and 27% following the R4F program.

#### 3.1.5. Sleep

**Figure 6:**
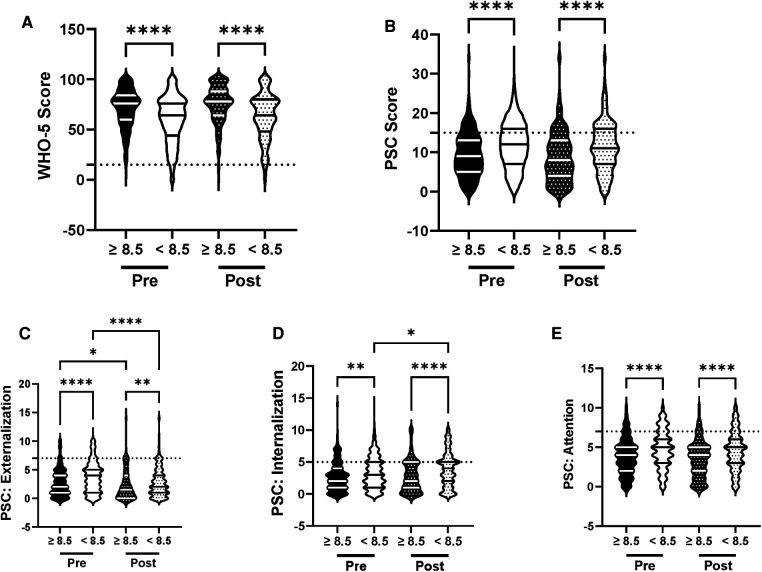
Impacts of sleep on psychosocial wellness and effectiveness of the R4F program. (**A**) Distribution of WHO-5 well-being scores in a violin plot. Dashed horizontal line shows the critical risk cutoff of 50 points with lower scores indicating lower well-being. (**B**) Distribution of PSC-17-Y composite well-being scores with a critical risk cutoff of 15 points. (**C–E**) PSC-17-Y subscores of attention, internalization, or externalization on violin plots with critical risk cutoffs of 7 points, 5 points, and 7 points, respectively. Higher scores indicate lower well-being. Within each violin plot the median and 25% and 75% quartiles are shown. Asterisks denote significance difference between groups based on a Kruskal-Wallis One-way ANOVA with a Dunn's multiple comparison test based on ranks (*<0.05, **<0.01, ****<0.0001). Pre-program, *N* = 315 for sleeping ≥8.5 h, *N* = 823 for sleeping <8.5 h. Post-program, *N* = 248 for sleeping ≥8.5 h, *N* = 564 for sleeping <8.5 h.

Because sleep is an important factor with regards to mental health and well-being we analyzed the influence of sleep on the association between the R4F program and mental health (40–42). Before as well as after the R4F program, those who slept at least 8.5 h had higher WHO-5 scores (Figure 6A) and lower PSC-17-Y composite scores (Figure 6B) compared to those who did not sleep at least 8.5 h. Before and after the R4F program PSC-17-Y externalization, internalization, and attention scores were higher in those who slept less than 8.5 h each night. Externalization PSC-17-Y sub-scores were associated with an improvement following the R4F program regardless of student sleep patterns (Figure 6C). Internalization sub-scores increased significantly following the R4F program in students who slept less than 8.5 h each night (Figure 6D) while the attention sub-score differences were unchanged following the R4F program (Figure 6E). The relative risk of developing psychosocial disorders was elevated in those who did not sleep 8.5 h or more each night as determined by WHO-5 (Figure 10A) and PSC-17-Y (Figure 10B) before and after the R4F program. Further, before the R4F program 28% slept 8.5 h or more each night, which was unchanged following the program being 31%.

#### 3.1.6. Physical activity levels

**Figure 7:**
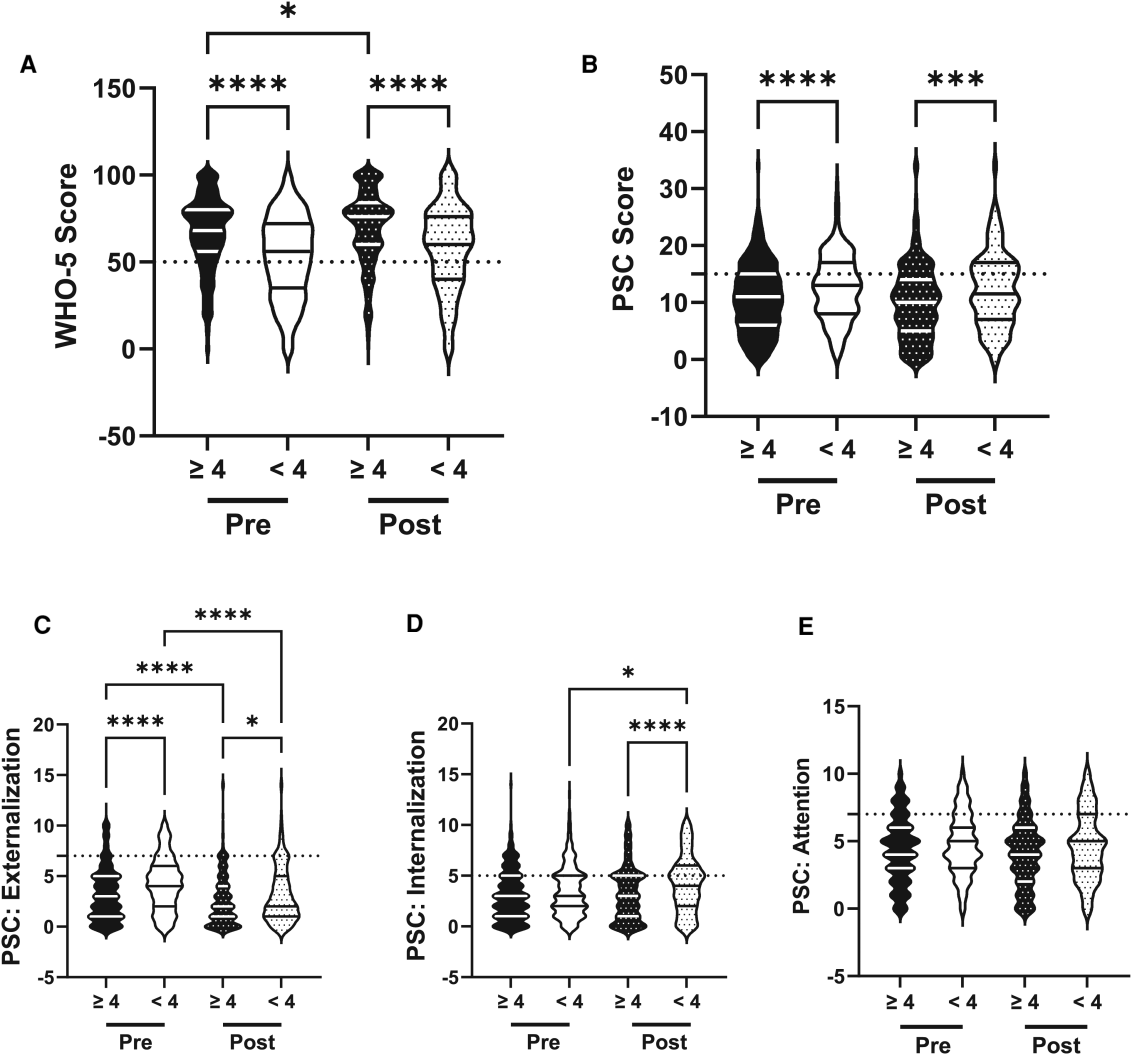
Impacts of physical activity on psychosocial wellness and effectiveness of the R4F program. (**A**) Distribution of WHO-5 well-being scores in a violin plot. Dashed horizontal line shows the critical risk cutoff of 50 points with lower scores indicating lower well-being. (**B**) Distribution of PSC-17-Y composite well-being scores with a critical risk cutoff of 15 points. (**C–E**) PSC-17-Y subscores of attention, internalization, or externalization on violin plots with critical risk cutoffs of 7 points, 5 points, and 7 points, respectively. Higher scores indicate lower well-being. Within each violin plot the median and 25% and 75% quartiles are shown. Asterisks denote significance difference between groups based on a Kruskal-Wallis One-way ANOVA with a Dunn's multiple comparison test based on ranks (*<0.05,, ***<0.001, ****<0.0001). Pre-program, *N* = 847 for ≥4 days/week, *N* = 290 for <4 days/week. Post-program, *N* = 609 for ≥4 days/week, *N* = 202 for <4 days/week.

We analyzed whether being physically active influenced the efficacy of the R4F program to improve mental health and psychosocial well-being because of the known associations between physical activity and mental health (6, 8, 18). Analysis of pre-program surveys revealed that students who exercised for 4 or more days each week had higher baseline WHO-5 well-being scores (Figure 7A) and lower PSC-17-Y (Figure 7B) scores than those who exercised less. Further, those who exercised less than 4 days each week had a higher relative risk of developing psychosocial disorders as measured through WHO-5 (Figure 10A) and PSC-17-Y (Figure 10B).

The significant differences in WHO-5 and PSC-17-Y scores associated with physical activity were further reflected in post-program results. Interestingly, participation in the R4F program was associated with an increase in the WHO-5 composite scores of those who exercised at least 4 days a week, which remained higher compared to those who exercised less. Participation in the R4F program was not associated with a change in the PSC-17-Y general composite score in either group (Figure 7B). Those who exercised at least 4 days a week had a significant decrease in externalization scores following participation in the R4F program (Figure 7C). Further analysis into the PSC-17-Y sub-scores revealed that internalization and externalization significantly increased in those who did not exercise at least 4 days a week (Figures 7C,D). There was no significant change in attention sub-scores following participation in the R4F program (Figure 7E). The relative risk of developing psychosocial disorders as determined by WHO-5 (Figure 10A) and PSC-17-Y (Figure 10B) also remained elevated in those students who exercised less than 4 days per week. Moreover, the proportion of students who exercised at least 4 days a week was 75% before and after participation in the R4F program.

#### 3.1.7. Screen time

**Figure 8:**
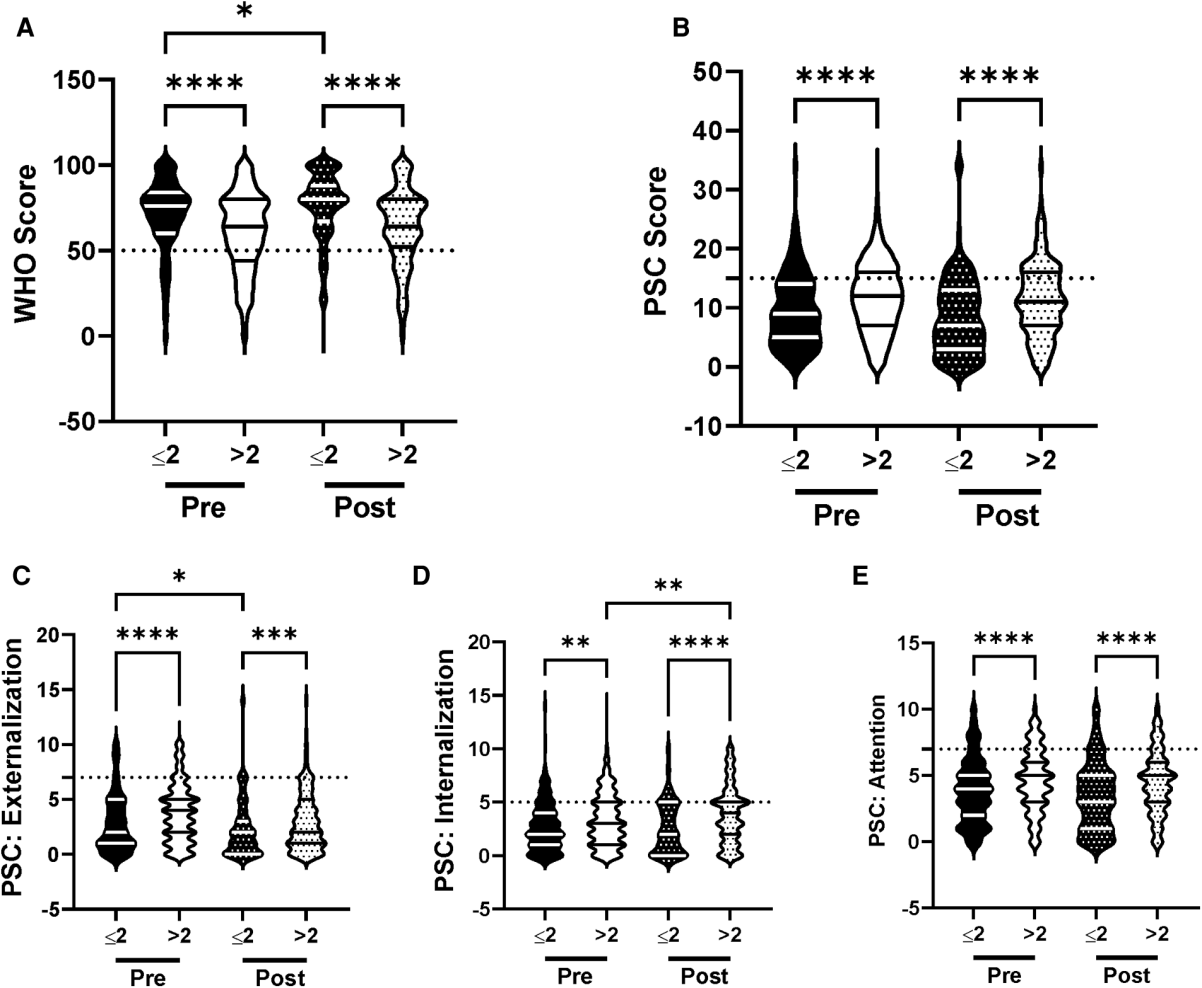
Impacts of screen time on psychosocial wellness and effectiveness of the R4F program. (**A**) Distribution of WHO-5 well-being scores in a violin plot. Dashed horizontal line shows the critical risk cutoff of 50 points with lower scores indicating lower well-being. (**B**) Distribution of PSC-17-Y composite well-being scores with a critical risk cutoff of 15 points. (**C–E**) PSC-17-Y subscores of attention, internalization, or externalization on violin plots with critical risk cutoffs of 7 points, 5 points, and 7 points, respectively. Higher scores indicate lower well-being. Within each violin plot the median and 25% and 75% quartiles are shown. Asterisks denote significance difference between groups based on a Kruskal-Wallis One-way ANOVA with a Dunn's multiple comparison test based on ranks (*<0.05, **<0.01, *** 0.001, ****<0.0001). Pre-program, *N* = 269 for ≤2 h/day, *N* = 873 for >2 h/day. Post-program, *N* = 218 for ≤2 h/day, *N* = 595 for >2 h/day.

We then analyzed the influence of screen time on the efficacy of the R4F program as current literature suggests that spending more than 2 h a day on electronic devices has a negative influence on psychosocial well-being (43). Before the R4F program students who exceeded the 2-hour limit had significantly lower WHO-5 composite scores (Figure 8A) and significantly higher PSC-17-Y composite scores (Figure 8B) as well as increased sub-score values for internalization, externalization, internalization, and attention (Figures 8C,D,E). Those who spent more than 2 h a day on electronic devices had increased relative risk of developing psychosocial disorders as measured through WHO-5 (Figure 10A) and PSC-17-Y (Figure 10B).

The significant differences in WHO-5 and PSC-17-Y scores between those who did and did not exceed the 2-hour threshold remained following the R4F program (Figures 8A,B). What is more, students who had less than 2 h of screen time showed a significant increase in their WHO-5 composite well-being scores after participation in the R4F program (Figure 8A). Participation in the R4F program was not associated with a change in the PSC-Y-17 well-being scores in either group of students (Figure 8B). Independent of the amount of screentime there was a decrease in the PSC-17-Y externalization sub-scores after the program (Figure 8C). Students who exceeded the recommended limit of daily screen use experienced an increase in internalization after the R4F program (Figures 8D). There was not any significant change in the PSC-17-Y attention sub-scores after the R4F program (Figure 8E). The relative risk of developing psychosocial disorders also remained elevated following the R4F program based on WHO-5 (Figure 10A) and PSC-Y-17 (Figure 10B). The proportion of students that met the screentime recommendation was 24% before the R4F program, which was not altered after the program being 27%.

#### 3.1.8. Club engagement

**Figure 9:**
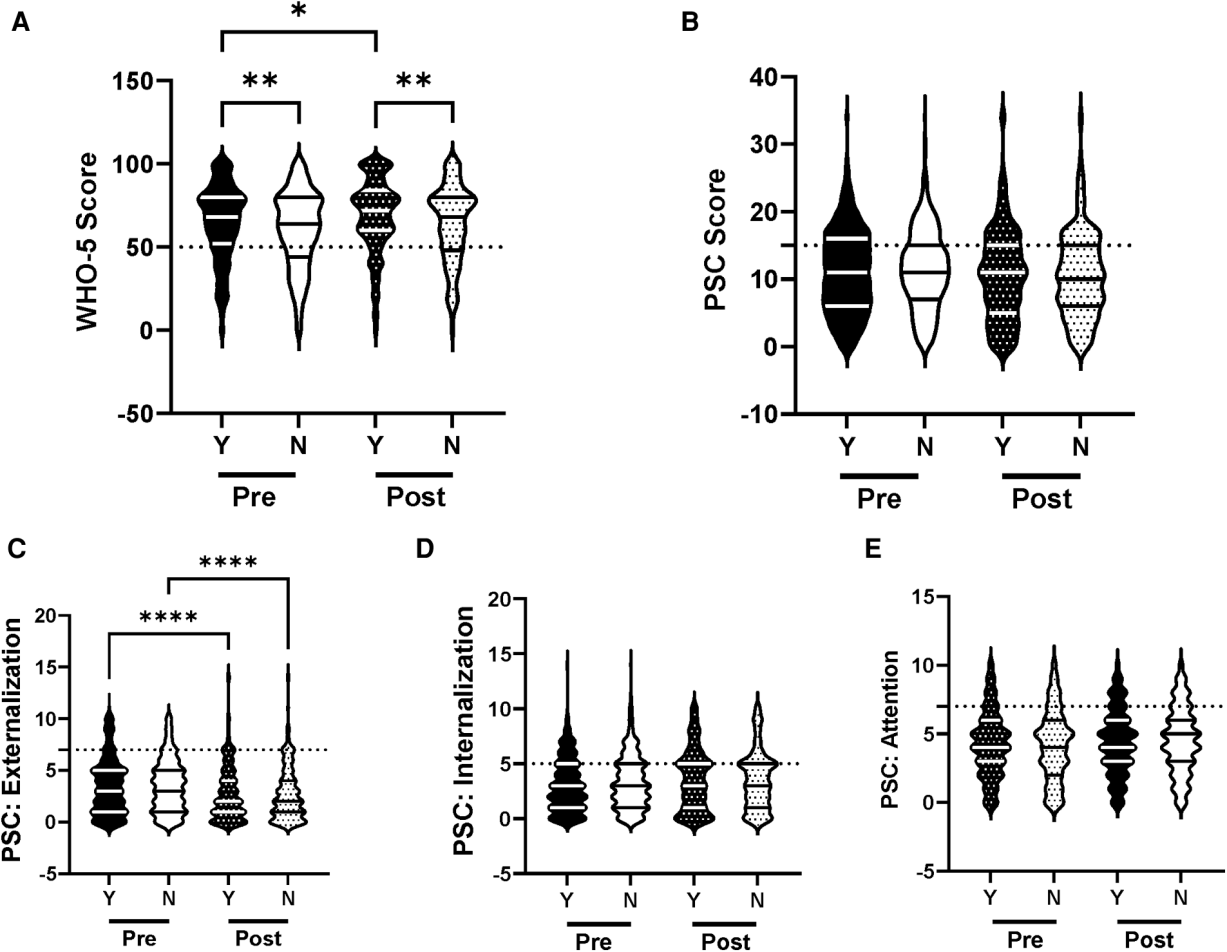
Impacts of participation in school activity on psychosocial wellness and effectiveness of the R4F program. (**A**) Distribution of WHO-5 well-being scores in a violin plot. Dashed horizontal line shows the critical risk cutoff of 50 points with lower scores indicating lower well-being. (**B**) Distribution of PSC-17-Y composite well-being scores with a critical risk cutoff of 15 points. (**C–E**) PSC-17-Y subscores of attention, internalization, or externalization on violin plots with critical risk cutoffs of 7 points, 5 points, and 7 points, respectively. Higher scores indicate lower well-being. Within each violin plot the median and 25% and 75% quartiles are shown. Asterisks denote significance difference between groups based on a Kruskal-Wallis One-way ANOVA with a Dunn's multiple comparison test based on ranks (*<0.05, **<0.01, ****<0.0001). Y, Involved; N, not involved. Pre-program, *N* = 641 for involved, *N* = 507 for uninvolved. Post-program, *N* = 436 for involved, *N* = 377 for uninvolved.

**Figure 10:**
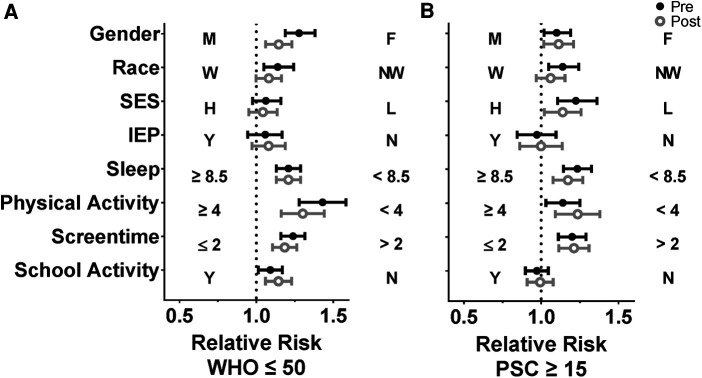
Impact of the R4F program on the relative risk of developing psychosocial disorders. (**A**) Relative risk based on WHO-5 scores below the critical cutoff of 50. (**B**) Relative risk based on PSC-17-Y scores 15 and above. As indicated on the figure, Relative risk was performed for the influence of male (M) vs. female (F) (gender), white identifying (W) vs. non-white identifying (NW) (race), low-income vs. medium/high income (SES), IEP (Y) vs. non-IEP (N), ≥8.5 h of sleep/night vs. <8.5 h of sleep/night, ≥4 days/weeks of physical activity vs. <4 days/weeks of physical activity (PA), ≤2 h/day screen time vs. >2 h/day of screen time, and involved in school activity (Y) vs. uninvolved in school activity (N) based on performing a Koopman asymptotic score (30).

We analyzed the influence of participation in school activities on the efficacy of the R4F program as current literature indicates that participation in school activities such as sports, clubs, and musical programs provide a positive influence on psychosocial well-being (44). Before the R4F program those who participated in school extracurriculars had significantly better WHO-5 scores relative to those who did not (Figure 9A). However, being in a school activity had no significant influence on composite PSC-Y-17 scores (Figure 9B) or on the externalization, internalization, or attention sub scores (Figures 9C,D,E). Those students who were not in clubs had a higher risk of developing psychosocial disorders as measured with the WHO-5 (Figure 10A) but not PSC-17-Y (Figure 10B).

Following the R4F program, WHO-5 scores increased significantly in students involved in school activities (Figure 9A) while PSC-17-Y composite scores were unchanged in either group of students. However, both involved and uninvolved students experienced a significant decrease in externalization PSC-17-Y sub-scores following the R4F program (Figure 9C) but there were no effects on internalization or attention (Figures 9D,E). Nor were there any differences in the sub score values between the two groups following the R4F program. The relative risk of developing psychosocial disorders remained improved relative to those who did not participate as determined by WHO-5 (Figure 10A) while the relative risk as determined by PSC-17-Y remained equivalent (Figure 10B). The proportion of students involved in school activities was 56% before the R4F program, which remained unchanged following program participation being 54%.

For all group comparisons pre- and post- intervention, a Cohen's *d* statistic was calculated. Nearly all aggregate changes in psychosocial metrics had a small effect size (Supplementary Table 1). Exceptions included female PSC-17-Y externalization scores (*d *= 0.5719, medium effect size), female PSC-17-Y internalization scores (*d *= −0.4303, medium effect size), and PSC-17-Y externalization scores for students who were active less than 4 days a week (*d *= 0.3901, medium effect size) (Supplementary Table 1).

## 4. Discussion

Our study examined the impact of a scholastic cycling physical education program on a few key factors. Analysis of the R4F surveys confirms the hypothesis that a middle school cycling education program is associated with positive impacts on student psychosocial well-being. Self-reported WHO-5 and the PSC-17-Y scores in an American adolescent population during the COVID-19 pandemic improved on aggregate after the R4F program. The scores were also stratified based on several factors. The primary finding of this stratified analysis was that the relative risk of developing psychosocial disorders was reduced in males, white students, those from high socioeconomic status families, those who get adequate sleep, those who are more active, those who have reduced screentime, and those who are involved in school activities, both before and after participating in the R4F program. Students who were involved in an IEP program did not have increased risk. Participating in the R4F program was generally associated with improved psychosocial measures, and had a positive effect in females, non-white students, students who are active, those who have reduced screentime, and those who are involved in school activities.

We first considered the general impact of the R4F program on WHO-5 and PSC-17-Y values, independent of any other factors. The finding that roughly a quarter of the pre-program respondents were below the critical thresholds in both the WHO-5 and PSC-17-Y metrics of psychosocial well-being and were at risk of depressive symptoms is in line with a recent meta-analysis (45). The discovery that participants in the R4F program saw improvements in both WHO-5 and PSC-17-Y metrics, and in the sub-scores of the PSC-17-Y for attention, externalization, and internalization reflect the consensus that physical education and school sport programs improve personal and social development and promote psychosocial competence (46). Our results also corroborate the fundamental concept that cycling contributes to improved mental health and well-being in adolescents (47). Indeed, the results are encouraging as they indicate that the R4F cycling program can be an effective tool for increasing the mental health of middle school students. The results are also exciting as the program improved the mental health and well-being of middle school students during COVID-19, a time of an unprecedented mental health crisis.

### 4.1. Riding for focus outcomes in the context of various risk factors

The finding that males had improved psychological well-being relative to females is consistent with a large body of research suggesting that females are more at-risk in terms of psychosocial outcomes (33, 34, 48). Although we did not collect surveys before the COVID-19 pandemic, the gender-related differences may have been exacerbated by the pandemic as the available literature indicates that adolescent females experienced greater mental health declines due to COVID-19 as compared to their male counterparts (49, 50). The data showing that female WHO-5 well-being increased following participation in the R4F program is encouraging and suggests cycling may be a promising way to support the mental health of female middle-school students, even though their scores did not reach the same level as male subjects. The worsening of the internalization but improvement in externalization sub-scores for females was surprising. By age 14 girls are more likely to internalize stress and anxiety than males (51). These diverse effects of the R4F program on the sub-scores indicate that the R4F program may need to be modified to optimize its efficacy in both male and female students, but further research is needed to better understand the potential driving factors behind this result.

The disparity in psychological well-being between white and non-white students corroborates current literature, which illustrates that minority racial groups have worse psychosocial well-being and have experienced a greater decline in psychosocial health during the COVID-19 pandemic as compared to white individuals (52). The positive impact of the R4F program on psychosocial well-being based on WHO-5 in non-white students is encouraging and supported by studies showing that physical activity programs can positively influence the psychosocial well-being of ethnic minorities, particularly those from low-income families (53). The improvement in externalization following the R4F program in both white and non-white students is promising and suggests that the program may help students who have various conduct and related disorders.

The varied findings regarding the impact of socioeconomic status on adolescent mental health and wellness are illustrative of the complexities associated with deriving associations based on SES. The finding that psychosocial well-being as determined by PSC-17-Y was lower in socioeconomically disadvantaged students was expected and follows recent studies (54, 55). However, that markers of depression were unaffected does not follow those reports, but rather is in line with current theories that a person's social support structure along with other social stresses influence the relationship between SES and adolescent depression (56, 57).

The lack of appreciable improvements in psychosocial well-being after participation in the R4F program for students who were socioeconomically disadvantaged follows recent findings that indicate exercise programs in low-income youth are largely ineffective (58). We cannot discount the potential that increased stressors due to the COVID-19 pandemic mitigated the influence of the R4F program in the low SES group (3, 45, 56). The positive association of the R4F program on externalization in low and high SES groups is an encouraging finding. Still, the lack of improvement in students from higher socioeconomic status following the R4F program on adolescent mental health and wellness is somewhat of a surprise given the primary positive effect of the study. Potentially this lack of effect may reflect the relatively small sample size in the SES subgroup. Socioeconomically disadvantaged students may also benefit more from multidimensional programs that combine the R4F program with other lifestyle programs such as those focused on improving nutrition (59), dental hygiene (60), or social interaction (56, 57, 61).

Students with an Independent Education Plan appeared to be protected, having equivalent risk of depressive, attention, and other psychosocial issues measured in our study as compared to students who did not have Independent Educational Plans. This finding is especially interesting as a recent study shows that during COVID-19, students who have attention deficit/hyperactive disorder experienced worsened psychosocial well-being (62, 63) and that physical activity helps mitigate ADHD symptoms (64). Albeit students with ADHD are only a subset of the diverse student population who have IEP programs. The increases in the WHO-5 scores in IEP students following the R4F program is compelling as it suggests those students have a decrease in depressive symptoms. Taken together, the findings support the premise that IEP programs afford students a measure of protection against psychosocial disorders. This posit warrants further exploration given the diversity of the student population who have IEP plans and their needs.

Sleep was critically important to psychosocial well-being. The finding that students who did not meet the CDC-recommended 8.5 h of sleep per night had worse baseline psychosocial well-being values across the various domains was expected. Indeed, a lack of sleep is a positive predictor of depressive symptoms and many individuals with depressive symptoms experience a lack of sleep (40, 42). The general failure of the R4F program to improve psychosocial well-being in students who did or did not meet the 8.5-hour sleep threshold is interesting and supports the importance of sleep in maintaining mental health and well-being. The improvement in externalization in all students is an encouraging finding, but this is offset by the worsening in internalization in those students who sleep less than 8.5 h each night. Thus, it is remains unclear if the R4F program can positively influence sleep patterns and the associations with psychosocial well-being. Resolving this question will be a future aspect to the work.

We were surprised that the R4F program selectively improved the psychosocial well-being of those students who self-identified as having higher physical activity levels. The finding that the R4F program was not associated with improved mental health in students who had low activity levels is disheartening, especially as a recent study illustrated that there was a decrease in the physical activity of adolescents due to COVID-19 pandemic-related restrictions (65). While we do not have any direct data from the schools we examined, COVID-19 restrictions may have compounded the disparity between more and less active students as social distancing and lockdown restrictions may have reduced or canceled training sessions. This would be especially prominent in students who participate in team sports or other extracurricular activities such as orchestra, where the lack of interaction reduces their ability to exercise and socialize. To better clarify the role of varying physical activity levels on psychosocial outcomes, future R4F programs could benefit from employing objective and quantifiable measures of physical activity levels and exploring program outcomes now that students no longer face pandemic related lockdowns.

The analyses regarding screentime use and the impact of the R4F program were revealing. The high proportion of students in our study who did not meet CDC screentime recommendations parallels studies showing that screen time use during COVID-19 increased appreciably, up to over 7 h per day (66). Interfaced with this, the finding that students had worse WHO-5 and PSC-17-Y scores before the cycling program corroborates other studies that illustrate more time spent on electronic devices worsens psychosocial well-being (67). Critically, the R4F program did not impact the percentage of kids who met the CDC standards, and the program did not counteract the negative impact of too much screen time. Moreover, we find it interesting that the R4F program selectively improved WHO-5 scores in those students who used their devices less than 2 h a day, which suggests that those who use devices longer each day get less mental-health benefit from doing physical activity.

The finding that students engaged in school activities such as clubs, sports, and music programs had better baseline psychosocial well-being scores as compared to uninvolved students is encouraging. Our studies are backed by current literature indicating that involvement in school-structured activities improves psychosocial maturity and well-being in adolescents (68). Interestingly, students who were involved in school club programs reported even higher levels of psychosocial well-being after participating in the R4F program compared to before participating, paralleling those students who had reduced screentime, or were more physically active. Notably, our findings may be skewed by the COVID-19 pandemic as the uninvolved students may have become more isolated because of pandemic restrictions when compared to previous school years. Thus, it is important for us to determine whether the R4F program will elicit similar improvements in psychosocial well-being in students who are not engaged in other school activities once they have a typical school year without COVID-19 related restrictions.

## 5. Limitations to the study

There are several limitations to the current study. The anonymous nature of the surveys that were used did not allow for within student, matched analyses. The current study also did not have control groups of students who did not receive the program, while the effect size was small. These issues will be overcome in future years with access to paired responses from individuals, by having appropriate control groups who do not receive the program, and by having subjects from a larger number of schools. Ongoing R4F programs are also more diverse, overcoming the narrowness of the racial demographics used in the current study. The current study was performed during the middle of the pandemic, which presented a confounder due to potentially declining levels of mental health and wellbeing. We look forward to collecting data from students in a post-pandemic world who have reduced stress, anxiety, and health risk. Future studies would benefit from a more nuanced and consistent method of stratifying the socioeconomic status of the students' families as compared to the enrollment in school lunch programs that have varied cutoffs that depend on family size and can vary from state to state. This is an important consideration as obtaining more precise income details may reveal hidden aspects regarding program effectiveness; trends that may help shape R4F curriculum and implementation to maximize the benefit for students from low-income families. Our study also only examined the acute effects of the program and did not examine the long-term impact of the R4F program on participant psychological well-being or their tendency to make healthy lifestyle choices. While some program effects may decline following the program, one might also see improvements for those who continue to ride bikes and exercise regularly. Lastly, the current surveys provide only limited information as they do not interrogate other lifestyle pillars. While exercise alone has proven to be effective in improving mental health, adopting changes to diet, sleep, and avoidance of risky substances positively influences mental well-being (69).

## 6. Conclusions

Our results indicate that participation in a school-based cycling intervention program has the capacity to improve psychosocial well-being in adolescents aged 11–14. Our results also indicate that the R4F program is associated with improved psychosocial outcomes that are negatively impacted by a number of well-established modifiable and non-modifiable risk factors. Though the R4F program on its own does not fulfill CDC recommendations for daily physical activity, student results demonstrate that program participation was still associated with improvement in youth mental health and well-being and a positive physical education experience.

### 6.1. Future directions

Despite limitations, we were able to use program evaluation data to both demonstrate feasibility of implementing a program under difficult conditions (COVID-19) and show encouraging program outcomes that are in-line with previously published studies. Moving forward, we propose several recommendations for future studies to increase the validity of program outcomes. First, future studies should make use of a control group and employ parallel controlled trial design to assess causation. In the context of continued surveillance and monitoring, it is recommended that participant data is tracked individually, along with being examined in aggregate. Additionally, to provide the broadest applicability it would be beneficial for the study populations to match US demographic trends more closely. Further, longitudinal studies will allow us to explore if participants continue to ride bicycles, build an active lifestyle, and examine the long-term effects of the R4F program on adolescent health and well-being. One additional consideration, even at this early stage of our understanding, is that providing culturally grounded versions of R4F could improve program efficacy (70).

## Data Availability

The raw data supporting the conclusions of this article will be made available by the authors, without undue reservation.
