# Supplementary material for: Middle school cycling program is associated with improved mental health and wellbeing in adolescents during COVID-19
Source: Front Sports Act Living. 2023 Oct 12;5:1255514. doi: 10.3389/fspor.2023.1255514 (PMC10600462; doi:10.3389/fspor.2023.1255514)
Supplement: Supplementary file 1 [file Table1.pdf]

**Supplemental Table 1<br>Results of Multivariate Analysis**								
**Category**	**Pre**		**Post**					
	**Mean**	**SD**	**Mean**	**SD**	**P-Value**	**Sig.**	**Cohen's *d***	**Effect Size**
**General**	n = 1148		n = 815					
**WHO-5**	62.88	22.88	66.60	22.65	0.0003	***	0.1634	Small
**PSC-17-Y General**	11.24	6.029	10.55	6.475	0.0051	**	0.1103	Small
**PSC-17-Y Extern.**	3.538	2.654	2.789	2.661	<0.0001	****	0.2818	Small
**PSC-17-Y Intern.**	3.213	2.451	3.539	2.709	0.0215	*	-0.1262	Small
**PSC-17-Y Attention**	4.487	2.418	4.226	2.478	0.0213	*	0.1066	Small
**Gender: Male**	n = 611		n = 424					
**WHO-5**	68.69	20.21	70.37	21.22	0.8563		0.0811	Small
**PSC-17-Y General**	10.45	5.984	9.849	6.540	0.2973		0.0959	Small
**PSC-17-Y Extern.**	3.046	2.469	2.976	2.755	>0.9999		0.0268	Small
**PSC-17-Y Intern.**	3.209	2.430	2.941	2.601	0.1609		0.1065	Small
**PSC-17-Y Attention**	4.198	2.394	3.932	2.415	0.5368		0.1106	Small
**Gender: Female**	n = 468		n = 335					
**WHO-5**	57.42	23.32	64.23	22.25	0.0003	***	0.2988	Small
**PSC-17-Y General**	11.61	5.836	10.97	6.279	0.4948		0.1056	Small
**PSC-17-Y Extern.**	3.925	2.678	2.442	2.505	<0.0001	****	0.5719	Medium
**PSC-17-Y Intern.**	3.024	2.401	4.119	2.681	<0.0001	****	-0.4303	Medium
**PSC-17-Y Attention**	4.665	2.393	4.406	2.478	0.7648		0.1063	Small
**Race: White**	n = 733		n = 500					
**WHO-5**	63.81	22.63	67.21	22.16	0.0700		0.1518	Small
**PSC-17-Y General**	10.88	6.089	10.22	6.181	0.5386		0.1076	Small
**PSC-17-Y Extern.**	3.412	2.679	2.530	2.428	<0.0001	****	0.3450	Small
**PSC-17-Y Intern.**	2.997	2.389	3.462	2.720	0.0347	*	0.1817	Small
**PSC-17-Y**	4.472	2.444	4.226	2.476	0.8703		0.1000	Small

**Attention**								
**Race: Non White**	n = 415		n = 315					
**WHO-5**	61.24	23.24	65.64	23.42	0.0431	*	0.1886	Small
**PSC-17-Y General**	11.87	5.877	11.09	6.893	0.0700		0.1218	Small
**PSC-17-Y Extern.**	3.761	2.597	3.200	2.952	0.0012	**	0.2018	Small
**PSC-17-Y Intern.**	3.593	2.516	3.660	2.690	>0.9999		-0.0257	Small
**PSC-17-Y Attention**	4.513	2.374	4.225	2.485	0.3483		0.1185	Small
**SES: Qualify**	n = 336		n = 251					
**WHO-5**	63.73	24.62	66.09	24.92	>0.9999		0.0953	Small
**PSC-17-Y General**	11.69	5.991	11.08	7.273	0.3003		0.0916	Small
**PSC-17-Y Extern.**	3.705	2.710	3.112	3.023	0.0041	**	0.2066	Small
**PSC-17-Y Intern.**	3.458	2.461	3.586	2.935	>0.9999		-0.0473	Small
**PSC-17-Y Attention**	4.711	2.392	4.378	2.613	0.3883		0.1329	Small
**SES: Do Not Qualify**	n = 391		n = 270					
**WHO-5**	64.30	22.20	68.46	21.11	0.1666		0.1920	Small
**PSC-17-Y General**	10.37	5.778	9.548	6.066	0.4646		0.1388	Small
**PSC-17-Y Extern.**	3.220	2.550	2.481	2.426	0.0007	***	0.2969	Small
**PSC-17-Y Intern.**	2.941	2.308	3.211	2.583	>0.9999		-0.1102	Small
**PSC-17-Y Attention**	4.207	2.389	3.856	2.458	0.5652		0.1448	Small
**IEP: Yes**	n = 126		n = 102					
**WHO-5**	67.49	21.84	74.51	23.60	0.0325	*	0.3087	Small
**PSC-17-Y General**	11.12	6.058	10.57	7.709	>0.9999		0.0793	Small
**PSC-17-Y Extern.**	3.492	2.727	3.167	3.209	0.7896		0.1091	Small
**PSC-17-Y Intern.**	3.278	2.478	3.353	2.852	>0.9999		-0.0281	Small
**PSC-17-Y Attention**	4.349	2.531	4.049	2.694	>0.9999		0.1148	Small
**IEP: No**	n = 359		n = 281					
**WHO-5**	64.07	23.18	66.09	22.84	>0.9999		0.0878	Small

**PSC-17-Y General**	10.56	6.004	10.55	6.427	>0.9999		0.0016	Small
**PSC-17-Y Extern.**	3.343	2.570	3.039	2.800	0.2704		0.1131	Small
**PSC-17-Y Intern.**	2.900	2.318	3.445	2.741	0.1572		-0.2147	Small
**PSC-17-Y Attention**	4.320	2.434	4.064	2.367	>0.9999		0.1066	Small
**Sleep: ≥ 8.5 hrs/day**	n = 315		n = 248					
**WHO-5**	70.92	19.55	75.08	19.30	0.0921		0.2142	Small
**PSC-17-Y General**	9.562	5.790	8.766	6.457	0.5763		0.1298	Small
**PSC-17-Y Extern.**	2.905	2.457	2.440	2.739	0.0220	*	0.1787	Small
**PSC-17-Y Intern.**	2.790	2.362	2.819	2.519	>0.9999		-0.0118	Small
**PSC-17-Y Attention**	3.867	2.320	3.508	2.357	0.6843		0.1535	Small
**Sleep: < 8.5 hrs/day**	n = 823		n = 564					
**WHO-5**	59.74	23.30	62.93	22.89	0.1101		0.1381	Small
**PSC-17-Y General**	11.93	5.991	11.38	6.315	0.2975		0.0894	Small
**PSC-17-Y Extern.**	3.793	2.688	2.954	2.615	<0.0001	****	0.3164	Small
**PSC-17-Y Intern.**	3.385	2.471	3.872	2.726	0.0139	*	-0.1872	Small
**PSC-17-Y Attention**	4.748	2.411	4.555	4.555	0.7612		0.0530	Small
**Physical Activity: ≥ 4 days/wk**	n = 847		n = 609					
**WHO-5**	66.41	21.47	69.52	21.30	0.0432	*	0.1454	Small
**PSC-17-Y General**	10.74	6.075	9.998	6.368	0.0874		0.1192	Small
**PSC-17-Y Extern.**	3.269	2.618	2.644	2.636	<0.0001	****	0.2379	Small
**PSC-17-Y Intern.**	3.092	2.445	3.259	2.652	>0.9999		-0.0655	Small
**PSC-17-Y Attention**	4.379	2.465	4.095	2.450	0.2491		0.1156	Small
**Physical Activity: < 4 days/wk**	n = 290		n = 202					
**WHO-5**	52.48	23.64	57.82	24.21	0.1002		0.2232	Small
**PSC-17-Y General**	12.58	5.706	12.21	6.493	>0.9999		0.0605	Small

**PSC-17-Y Extern.**	4.266	2.618	3.219	2.748	<0.0001	****	0.3901	Medium
**PSC-17-Y Intern.**	3.517	2.454	4.232	2.761	0.0334	*	-0.2737	Small
**PSC-17-Y Attention**	4.797	2.267	4.624	2.499	>0.9999		0.0725	Small
**Screentime: ≤ 2 hrs/day**	n = 873		n = 595					
**WHO-5**	70.68	20.94	76.11	20.17	0.0211	*	0.2641	Small
**PSC-17-Y General**	9.550	6.178	8.413	6.523	0.2615		0.1790	Small
**PSC-17-Y Extern.**	2.881	2.637	2.307	2.676	0.0276	*	0.1206	Small
**PSC-17-Y Intern.**	2.799	2.387	2.619	2.486	>0.9999		0.0739	Small
**PSC-17-Y Attention**	3.870	2.448	3.486	2.558	0.6896		0.1534	Small
**Screentime: > 2 hrs/day**	n = 269		n = 218					
**WHO-5**	60.45	22.95	63.17	22.36	0.2675		0.1201	Small
**PSC-17-Y General**	11.78	5.889	11.37	6.270	0.5541		0.0674	Small
**PSC-17-Y Extern.**	3.746	2.629	2.971	2.638	<0.0001	****	0.2943	Small
**PSC-17-Y Intern.**	3.347	2.460	3.886	2.708	0.0026	**	-0.2084	Small
**PSC-17-Y Attention**	4.687	2.381	4.509	2.386	0.9129		0.0747	Small
**Extracurricular Participation: Yes**	n = 641		n = 436					
**WHO-5**	64.85	22.58	69.00	21.97	0.0246	*	0.1863	Small
**PSC-17-Y General**	11.05	6.129	10.58	6.653	0.9035		0.0735	Small
**PSC-17-Y Extern.**	3.510	2.727	2.830	2.721	<0.0001	****	0.2496	Small
**PSC-17-Y Intern.**	3.112	2.472	3.539	2.784	0.1307		-0.1622	Small
**PSC-17-Y Attention**	4.429	2.414	4.265	2.391	>0.9999		0.0683	Small
**Extracurricular Participation: No**	n = 507		n = 377x					
**WHO-5**	60.39	23.03	63.73	23.14	0.1484		-0.1447	Small
**PSC-17-Y General**	11.47	5.898	10.55	6.278	0.0682		0.1510	Small
**PSC-17-Y Extern.**	3.574	2.560	2.743	2.600	<0.0001	****	0.3221	Small

**PSC-17-Y Intern.**	3.339	2.422	3.544	2.628	>0.9999		-0.0811	Small
**PSC-17-Y Attention**	4.560	2.423	4.209	2.546	0.2118		0.1412	Small

Asterisks found in the “Sig.” column denote significance difference between pre and post for the aggregate data (general) based on a Mann-Whitney U test (*<0.05, **<0.01, ***<0.001, ****<0.0001). For all subsequent groups, a Kruskal-Wallis One-way ANOVA with a Dunn’s multiple comparison test based on ranks (*<0.05, **<0.01, ***<0.001, ****<0.0001) was used. SES = socioeconomic status; IEP = Individualized Education Plan. Metrics include the World Health Organization-5 (WHO-5), the pediatric symptom checklist (PSC-17-Y), and its subsequent subscores of externalization (PSC-17-Y Extern.), internalization (PSC-17-Y Intern.) and attention (PSC-17-Y Attention).
